# The experience of bereaved cancer carers in rural and regional areas: The impact of the COVID-19 pandemic and the potential of peer support

**DOI:** 10.1371/journal.pone.0293724

**Published:** 2023-11-07

**Authors:** Natalie Winter, Kerry McKenzie, Danielle Spence, Katherine Lane, Anna Ugalde

**Affiliations:** 1 School of Nursing & Midwifery and Institute for Health Transformation, Deakin University, Geelong, Victoria, Australia; 2 Strategy and Support Division, Cancer Council Victoria, Melbourne, Australia; University of Technology Sydney, AUSTRALIA

## Abstract

**Background:**

Caring for someone with cancer during end of life care can be a challenging and complex experience. Those living in rural and regional areas are less likely to have local healthcare services and may be physically isolated. Even where support services such as respite do exist, they may be less likely to be accessed due to the time burden in travelling to services. This was compounded by the COVID-19 pandemic.

**Aim:**

To understand the potential benefits of peer support for bereaved carers of people with cancer from rural and regional locations during the COVID-19 period.

**Methods:**

Phone interviews were conducted with bereaved cancer carers living in rural and regional areas in Victoria. Semi-structured interviews were used, and participants were asked about their experience as a carer, bereavement and the potential for peer support. Interviews were audio recorded and transcribed verbatim; transcripts were coded and a thematic analysis was conducted.

**Findings:**

12 interviews were conducted. Carers were mostly female (85%) and were on average 58 years of age (range 42–71). Interviews lasted an average of 58 minutes (range 53–91 minutes). Three themes were derived from the data; 1) Supportive care needs while caring and the impact of COVID-19; 2) Isolation during bereavement compounded by the COVID-19 pandemic; and 3) Peer support requires flexibility to meet diverse needs.

**Conclusion:**

Peer support has potential to assist bereaved carers of people with cancer. A co-design approach may be beneficial for developing a flexible model for supporting and linking carers together.

## Introduction

Cancer is the leading cause of death worldwide with over 10 million reported deaths in 2020 [1]. Worldwide, Australia and New Zealand have the highest prevalence of cancer in part due to increased screening and detection [1].

Most people with cancer have a carer; a friend or family member who take on an extensive role inprovision of care [2]. Caring for someone with cancer can be a challenging and complex experience. Being a carer can result in increased levels of distress, anxiety or depression [3–5], which is often greater than the overall distress experienced by patients themselves [6]. Approximately 90% of people with advanced cancer have a family caregiver [2]. These carers can experience a higher number of supportive care needs, compared to carers looking after someone living with other stages of cancer [7]. Carers can experience pre-emptive grief during end of life care [8] and grief during bereavement immediately following death [9]. Bereavement can last for several years after the death of the patient [9]. There is a need for bereavement support for carers prior to end of life care.

Poor outcomes for carers are further compounded for those living in rural and regional areas as they are less likely to have services nearby including access to clinicians, respite, and support groups. Carers may be physically isolated, and where clinical services exist, they may be less likely to be drawn on due to time barriers or cultural appropriateness [10]. In Australia approximately 28% of the population live in rural and remote areas [11], with large geographical catchment areas for healthcare services [12]. The ability to coordinate care through tertiary healthcare provision with various levels of federal and state funding, presents a challenge for delivering care to rural and regional communities. In these communities the need for services may not outweigh the cost of implementing and maintaining programs [12], this is evident in palliative and bereavement care settings where inconsistenices exist in carers’ access to support [13]. As a result, there is a need to support carers in these communities in a cost-effective, easily accessible and sustainable manner.

COVID-19 has resulted in significant disruptions to cancer care including early screening, diagnosis, access to treatment and limited contact with health professionals [14, 15]. During the March 2020 to October 2021 period, Victoria experienced a series of preventative public health lockdowns to reduce COVID-19 transmission; the longest lockdown spanning 112 days. Restrictions included leaving your home for non-essential purposes and engaging in face-to-face contact with others [16].

Comparison studies between Victoria and other states in Australia indicate that prolonged lockdowns in Victoria led to increased feelings of distress, social isolation, and loss of work compared to other states [16]. As a result of these lockdowns, Australia had the second lowest percentage of positive test results worldwide [17]. Other studies have reported similar long-term impacts of COVID-19 on carers internationally [18], however, the impact on bereaved cancer carers who are from rural/regional settings remains unclear. COVID-19 has resulted in unique challenges for cancer patients and carers [4] and there is a desperate need for additional information and support for carers during these periods of heightened uncertainty [19]. Specifically within the cancer context additional uncertainties can include worries related to patients dying alone, the availability of vaccines, and the community spread of the virus [6].

During the COVID-19 period, caregivers in rural settings reported substantial increases in caregiver burden at a rate more than double that of their metropolitan counterparts [20]. People in rural areas were particularly dependent on telehealth to deliver and monitor health, which at times created a barrier in involving carers as part of the healthcare team [21]. In other chronic illnesses such as dementia, social networks were a source of support for carers in rural areas, and cessation of these supports resulted in increased isolation [22, 23]. Evidence continues to emerge providing insight into how COVID-19 has impacted carers’ wellbeing, for example, one study indicates that elderly carers in rural areas have higher levels of depression compared to younger carers, or those living in metropolitan areas [24]. Recommendations suggest that it is vital to provide guidance, information and services where possible to alleviate the burden on carers [19]. Further, it is crucial for carers to utilise and foster existing social connections and extended family to provide and receive support when access to service based care is limited [19].

In addition to leaning on existing social networks, peer support is another type of accessible and cost-effective intervention. Peer support can differ from other social support networks as participants of peer support can receive advice related to medical care, emotional support, recommendations for support services, and can be accessed as required over extended periods of time [25]. Peer support is a vital part of supportive care and enables patients and carers to feel less isolated, and learn coping strategies and share information with someone who has a lived experience similar to their own [26, 27]. It also has benefits for the peer volunteer as many describe a sense of satisfaction with being able to give back to others with a cancer diagnosis [28]. Peer support can be beneficial for cancer carers throughout the caring trajectory and can support a variety of different key topics including disease information, treatment, self care, and bereavement [29].

Despite the known benefits of peer support, there are limited options available for those who are caring for advanced cancer patients and into bereavement and, in particular for people living in rural and regional areas.

### Aim

To understand the potential benefits of peer support for bereaved carers of people with cancer from rural and regional locations during the COVID-19 period.

## Methods

### Research design

This project used qualitative descriptive methodology [30] using phone interviews with carers from regional and rural areas in Victoria.

### Setting and context

Participants were recruited from the Grampians region in Western Victoria, Australia, which covers almost 50,000 square kilometres with a population of almost 220,000 people. Local government areas Ararat, Ballarat, Golden Plains, Hepburn, Hindmarsh, Horsham, Moorabool, Northern Grampians, Pyrenees, West Wimmera and Yarriambiack were included [31].

Interviews were conducted over July–October 2021.

### Participants

Participants were bereaved informal carers defined as a family member or friends who met the following inclusion criteria:

- Adults over the age of 18,
- The ability to speak English without the aid of an interpreter,
- Carers who were bereaved and were the primary person provided support to someone living with advanced cancer
- Participants needed to be living within the target rural area

### Procedure

A variety of recruitment methods were utilised including advertising on websites, local newsletters, social media, and promotion of the study by clinicians at health services.

Interested carers initiated contact with the project manager (KM) who screened individuals for eligibility, completed informed consent and demographic questionnaires, and scheduled phone interviews. Phone interviews were conducted by NW and AU. Phone interviews were selected as the preferred method given the geographical distance between the interviewers and participants and travel restrictions during the COVID-19 pandemic.

### Data collection

Individual phone interviews were scheduled with carers. All interviews were semi-structured, participants responded to the same questions and prompts related to their experiences caring, the availability of support, and their thoughts around how a potential new peer support model could be designed and delivered. As interviews were conducted over July–October 2021 during the COVID-19 period, participants reflected on their experiences either before and/or during the COVID-19 lockdowns, however, no formal questions related to the impact of COVID-19 were asked. Interviews continued until data saturation occurred which was mutually agreed upon by two authors (NW and AU). Both interviewers are female PhD qualified academics with experience in qualitative research, and who had no prior relationships with carers involved.

### Analysis and reporting

Phone interviews were audio recorded and transcribed verbatim. We followed a similar coding process as described by Colorafi and Evans (2016) [32]. At the end of each interview overall topics were discussed between the two researchers (NW and AU). Transcripts were then read in full and key concepts were created into codes. An iterative process was used where new concepts from subsequent interviews were added and previous transcripts were re-coded to include new concepts. This provided as flexible coding framework that could be used in between each interview and to allow for proper assessment of data saturation. Results are presented below with accompanying quotes from participants. Numbers have been assigned to participants to demonstrate a breadth of perspectives represented in the results section. The numbers have been randomly assigned are not indicative of the order of interviews or any other features of participants.

## Results

### Demographics

12 carers were interviewed. The majority of carers were female (85%) and were on average 58 years of age (range 42–71), See Table 1 for the full demographics. Interviews lasted an average of 58 minutes (53-91minutes).

**Table 1:** Demographic characteristics of carers and healthcare professionals (N = 12).

	Mean (range)
Age	58 (42–71)
	Frequency (%)
Gender	
Female	11 (92)
Aboriginal or Torres Strait Islander	
No	12 (100)
Highest level of education	
University degree	6 (50)
Certificate or diploma	5 (42)
Communication devices used	
Landline telephone	3 (25)
Mobile phone	12 (100)
Desktop computer	4 (33)
Laptop	11 (92)
Tablet	7 (58)
Do you identify with the terminology ‘carer’	
Yes	10 (83)
Relationship to patient	
Spouse	6 (50)
Daughter	5 (42)
Father	1 (8)
Living with the person receiving treatment	
Yes	11 (92)
Type of cancer	
Breast	2 (17)
Prostate	2 (17)
Brain	1 (8)
Oesophageal	1 (8)
Non-small cell lung	1 (8)
Bowel	1 (8)
Acute myeloid leukaemia	1 (8)
Oral	1 (8)
Bile duct	1 (8)
Leukaemia	1 (8)
Length of caring period	
< 6 months	3 (25)
6–12 months	2 (17)
1–2 years	3 (25)
2+ years	4 (33)

### Findings

Findings were grouped into three themes; 1) Supportive care needs while caring and the impact of COVID-19; 2) Isolation during bereavement compounded by the COVID-19 pandemic, and; 3) Peer support requires flexibility to meet diverse needs.

#### Theme 1: Supportive care needs while caring and the impact of COVID-19

Some carers described looking after someone in end of life care prior to the COVID-19 pandemic. During these times carers’ could give and receive peer support informally. One carer retold the experience of receiving peer support informally while providing end of life care. He said that when the person he cared for was very unwell in hospital:

“A woman from the ward, she came up and just held me.” (C2).

He later noted how he was able to provide peer support for others:

“When a man collapsed–I thought–this is what I can do. This is how I can assist (the carer).” (C2)

With data collection occurring over July–October 2022, the impact of COVID-19 and the impact on caring and bereavement was discussed by all participants. Carers noted they were particularly affected by the impacts of lockdowns, although were unable to seek support from their social networks. Some reflected that the changes during end of life care were swift, from a large support network to an absence of visitors, though at times were able to acknowledge the positives of this:

“Wow (having her at home), what a privilege. We were so lucky to have her with us. Covid was new. For the first four weeks (of her illness) it involved a lot of her friends, jigsaws, cups of coffee. Then we couldn’t have anyone in our home. Things changed quickly.” (C12)

#### Theme 2: Isolation during bereavement compounded by the COVID-19 Pandemic

Carers described an abrupt end to support after the death of their loved one from formal support services and extended family members.

“There is that sense of abandonment by all these people who are helping and then they’re gone … from the Hospice people especially. You know, I understood that they would continue with a couple of visits or calls to me, but there hasn’t been anything at all.” (C13)

Some carers experienced the death of their loved one when there were no lockdown restrictions in place. During these times, the number of people able to attend events was still restricted by imposed capacity limits. Carers described that sudden recommencement of lockdowns affected funeral plans:

“We were only allowed 10 at the funeral [during lockdown] and when he first passed away, we were told we could have 31 at the funeral [outside of lockdown]. And actually, that was quite problematic because it’s a case of you’ve got to make an invitation list. And well, yeah, who do you invite? Who don’t you invite?” (C4)

One carer reflected on the challenges in having funerals and memorials postponed multiple times. She reflected on the process of planting trees alone, which had been planned as an activity for friends and family at a memorial service:

“[A community group] were planning an event for dad… It had to be cancelled again. It’ll be two years in March 2022 and we still won’t be able to do stuff. We couldn’t have the funeral. I thought we’d have a big tree planting day. I bought all these trees… I had to plant them, by myself.” (C3)

Some carers recognised that there was a gap in support during bereavement and the pandemic made it challenging to make and foster peer connections:

“I’ve being going to start a widowed people support group here in [regional town] for a while but with COVID, it’s a bit hard.” (C7)

#### Theme 3: Peer support requires flexibility to meet diverse needs

Overall there was a general consensus that peer support could play a role in supporting carers during and after caring for someone with cancer:

“Just have someone keep contacting you for a few months, absolutely (that would be helpful)… To let them know that they’re not alone.” (C9)

“[A bereavement group would be useful for] just, you know, in practical terms and in terms of the complexity of grieving… but there’s also…what I call grief confusion where you’re kind of on a mission to get it all sorted and yet at the same time, you’re absolutely bloody exhausted.” (C10)

Variance existed in how carers thought that peer support could be offered. Preference was often given for face-to-face formats, as this provided carers with the opportunity to form peer connections and provide the giving and sharing of mutual support. One carer reflected on the importance of being with others:

“To actually sit with someone who is going through it for the first time.” (C4)

Others felt that face-to-face may be too overwhelming during the heightened emotional period accompanying bereavement care:

“I don’t know about face-to-face… I was very good at bursting into tears regularly and so the face to face might be too confronting.” (C1)

To accommodate the diverse experiences that carers face, carers reported that having peer support available across multiple formats may be appropriate to meet their needs, particularly when they were unable to leave patients unattended:

“I would have thought that a telephone conferencing is probably quite good. I mean, for an occasional face to face and that would that fit in well with those feelings of not being able to leave your partner.” (C10)

The concept of an “informal community” resonated with carers where they could check in on each other on an ad hoc basis, and have the ability to organise and connect with each other through peer support channels:

“Have an option that if you’re needing more support and you’re comfortable with the peers that are around you and you’re comfortable with saying, hey I’m struggling today, can we catch up for a cuppa?” (C4)

In addition to emotional support, carers felt that their daily and practical needs while in the final stages of caring could be addressed through clinician chats or vignettes:

“If there was some sort of telephone service, if someone was really finding it hard, that that could talk to someone who understood about the caring role or could offer some tips… like a webinar type thing or a little video they could watch online that gives them ideas.” (C7)

Carers described that having support specifically within the rural and regional context was vital as experiences and unmet needs were unique compared to carers living in metropolitan areas:

“The country environment lends itself to other difficulties that the city would never ever have. Distance, lack of ability to support kids…I think a regional contact is actually going to provide more support” (C1)

## Discussion

In this study we described the experiences of bereaved cancer carers from rural and regional areas. The majority of participants were female, caring for a spouse, lived with the person with cancer, and identified as a carer. Key findings indicated that the COVID-19 pandemic placed a greater burden on some carers in their ability to receive and seek support both from healthcare professionals and their social networks during bereavement. Carers identified that peer support has a place in supporting carers, however, the mode of delivery and intended use in accessing peer support varied based on carers’ own needs and preferences.

Previous studies have described carers’ feelings of social isolation and reduced access to healthcare services during the COVID-19 pandemic [18]. Our study provides additional findings about the experiences of carers within regional and rural context, who rely more heavily on peer and community support than carers living in metropolitan areas [33]. As identified in this study, carers described that providing support to a loved one during end of life care was challenging due to the availability of services in rural and regional settings and the compounding impact of COVID-19 on the lack of available services. Further, opportunities to connect with others from peer or community groups were limited as they were inaccessible during lockdown and no online resources existed. Supporting carers in the community has been noted as a priority area of concern with recommendations on how to address carers’ own needs as well as integrating them into the healthcare team via telehealth [19]. The pandemic has seen an exponential uptake in the use of digital technologies in the cancer field. Telehealth has provided patients with the ability to remotely attend follow up appointments [34] and monitor symptoms for clinician review within the outpatient setting [35]. The role of technology in supporting carers throughout and beyond the pandemic is currently still under investigation [36]. However, restrictions imposed with COVID-19 lockdowns and inpatient care has seen strict limitations worldwide in the allowance of family and visitors to hospital appointments and admissions [37, 38]. As a result, the opportunity for carers to seek and receive support from healthcare professionals was reduced during bereavement. Carers in this study noted the lack of support available during end of life care and bereavement periods. How we can best deliver peer support to carers requires further exploration as society adapts to living with COVID-19, while also protecting those who are caring for people who are immunocompromised or terminally ill.

Clearer identification of carers in the community is needed to prompt clinicians to provide referrals to supportive resources. Where the term ‘carer’ is not adopted by family members, clinicans have described being unsure of which family members require assistance [39]. Identification with the term ‘carer’ may not apply to everyone in caring roles [40] and in some circumstances may be a gradual process which may be influenced by deterioration in patients’ health [39]. While 83% of participants in our study identified with the term ‘carer’, access to support was limited due to living in a rural location, highlighting the need for locally run supportive interventions such as peer support.

Carers described that peer support could be of benefit during bereavement. For many this was the giving and sharing of practical tips, emotional support, and understanding of someone else with a similar experience specifically from a rural or regional location. Peer support has similarly shown to be beneficial in the bereavement period for family members following sudden or unexpected death of a loved one [41], or following the death of a child with cancer [42].

There were a variety of preferences for the ideal peer support program that could be adopted in rural and regional areas, with a key preference for flexibility. Flexibility included the timing and frequency of peer support sessions, as well as the delivery of sessions either in face-to-face or online settings. Flexibility was preferred as this would allow carers to access support based on their individual needs and preferences, and is consistent with previous studies [43, 44]. Similarly, among carers of people living with dementia, face-to-face peer support remained a preference during lockdown restrictions due to the enjoyment experienced in group settings [44]. This suggests the need for blended models of peer support.

One suggestion to meet carers’ needs and ensure uptake of a future program is to use a co-design framework working with key stakeholders including carers, healthcare professionals, and hospice. Co-design can provide an opportunity to guide the design, modality and frequency of a peer support program, and previous research has highlighted that co-design can also be used to support project implementation [45], in particular within priority groups in the community [46].

The need to work with consumers to develop a specific rural and regional peer program is evident. Despite the potential use of technology to connect carers across Victoria, there was a preference that the peer support program be specific to carers from rural and regional locations due to the unique experiences of those living in the county. The notion that peer support differs between rural and regional and metropolitan settings has been described previously [47]. This suggests that geographical location of carers need to be taken into account when designing interventions to ensure that interventions meet the needs of all end users. A variety of rural peer support resources have been developed with consumers; the development of YouTube videos [48] found similar results where the needs of carers in rural and regional context are unique. And, in the United States of America, a coaching intervention for carers of people with advanced cancer was adapted to be delivered via telephone [49]. This study suggests that geographical challenges are an international challenge as is the need for digital based resources.

This study was conducted during the COVID-19 pandemic and when the study commenced it was unclear how much of an influence the pandemic would have on data collection. As a result no data were collected about the timing of the death of the patient. Many of the sample had been bereaved prior to COVID-19, but most noted an impact on their support networks and opportunity to seek ongoing bereavement care during the pandemic and associated lockdowns. To ensure uptake of future peer support programs, it may be beneficial to embed programs into existing services with routine referrals for carers from clinicians involved in patients’ care. A similar need for referral into existing services is required from General Practice doctors supporting carers in the community [50] and for carers of people within Veteran Affairs programs [51].

## Conclusion

The ability for bereaved cancer carers to find support was impacted during the COVID-19 pandemic, and carers were particularly isolated. Peers may help provide support for carers during bereavement. Peer support programs should be designed with stakeholders to ensure program uptake. Programs may need to be embedded into existing systems with routine referral to support caregivers in the active palliative care phase.
